# Epidemiology and Management of Open Fractures in a Tertiary Care Hospital in Nepal: An Observational Study

**DOI:** 10.31729/jnma.8860

**Published:** 2025-01-31

**Authors:** Sagar Maharjan, Arun Kumar Chaudhary, Rohit Shrestha, Pramit Ram Shrestha, Prabesh Gautam, Ashkal Basi, Manasil Malla, Shreedhar Prasad Acharya, Sushant Kumar Khadka

**Affiliations:** 1 Department of Orthopedics and Traumatology, Dhulikhel Hospital, Kathmandu University Hospital, Dhulikhel, Kavre, Nepal; 2 Department of Statistics, Nepal Commerce Campus, Tribhuwan University, Minbhawan, Kathmandu, Nepal; 3 Kathmandu University School of Medical Sciences, Dhulikhel, Kavre, Nepal; 4 Kathmandu Medical College, Sinamangal, Kathmandu, Nepal

**Keywords:** *gustilo classification*, *open fractures*, *resuscitation*, *trauma*

## Abstract

**Introduction:**

An open fracture is an orthopedic emergency that requires immediate resuscitation and stabilization. Understanding presentation and management patterns can help prepare in emergency settings. This retrospective descriptive cross-sectional study stated the demographic profile, management pattern, and seasonal distribution of patients presenting with open fractures.

**Methods:**

An observational cross-sectional study was conducted in a tertiary care center. Medical records of all patients with open fractures who underwent immediate orthopedic interventions between January 2021 and December 2023 were evaluated retrospectively after obtaining ethical approval from the Institutional Review Committee (Reference number: 29/24). Descriptive statistics were used to analyze data.

**Results:**

The study included a total of 133 cases of open fractures managed during the study period. Out of these, male patients were 104 (78.20%) and female patients 29 (21.80%), with a median age of 35.00 (25.00 - 42.00) years. Mode of injuries included road traffic accidents 53 (39.85%) and falls 32 (24.06%). Tibia fracture was seen in 56 (42.11%) cases, and spinal anesthesia was used in 52.63% of cases. External fixators 36 (27.07%) and intramedullary nails 29 (21.80%) were the primary surgical interventions.

**Conclusions:**

Male patients with open fractures were more in comparison to female patients. Road traffic accidents were the most common cause of injury. Gustilo III B fractures were the most frequent kind of injury with tibia most commonly involved. External fixator application was the most common procedure for immediate stabilization.

## INTRODUCTION

Open fractures are one of the common orthopedic emergencies and their management poses significant challenges, necessitating prompt resuscitation and stabilization to optimize the long-term outcome.^1^ Open fractures result due to high-energy trauma with more than half originating from either road traffic accidents (RTA) or falls from considerable heights. RTA rates have rapidly increased in low-income nations.^1^ The incidence of RTA in Nepal has increased from 11,747 in 2009/10 to 25,788 in 2019/20 which can suggest a similar rate of increase in cases of open fractures.^2^

There is limited information available regarding the epidemiological profile, demographics, and management strategies of the cases presenting with open fractures in Nepal. Understanding these factors could improve case management and may inform public health policies to prevent such injuries. This study hence aimed to study the epidemiology of open fractures presenting in a tertiary care hospital delineating the epidemologic characteristics and management strategies of open fractures.

## METHODS

This is an observational cross-sectional study carried out at Dhulikhel Hospital, which is a tertiary care center located in Kavre, Nepal. Retrospective data from January 2021 to December 2023 was collected from patients' records after ethical approval from Institutional Review Committee (Reference number: 29/24). Records of all patients who presented to the Dhulikhel Hospital with open fractures were included in the study. Records of patients with all other orthopedic emergencies, patients presenting with other orthopedic problems, duplicate records, and follow-up cases of open fracture cases were excluded. All the patients received immediate resuscitation, including primary and secondary surveys, intravenous antibiotics, wound irrigation, splinting, and definitive intervention. Total samplig was done. Data were retrieved from the record section and checked for completeness.

The descriptive statistical analysis was done with Microsoft Excel for Mac 2011 ver. 14.0.0. Categorical data were presented in numbers and percentages and continuous data were presented in terms of median and interquartile range (IQR) along with tables and charts as appropriate.

## RESULTS

**Figure 1:**
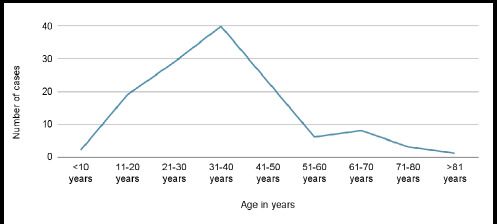
Age-wise distribution of patients with open fractures (n=133).

There were a total of 133 cases of open fractures that presented to Dhulikhel Hospital during the study period. The total number of male patients was 104 (78.20%) and female patients were 29 (21.80%), and the median age of the study cohort was 35.00 (25.00 - 42.00) years with 113 (84.96%) patients in their physically active period of life , between 11 to 50 years (Figure 1). The temporal variation of cases showed 45 (33.83%) cases in winter followed by 35 (26.32%) in spring (Table 1).

**Table 1:** Temporal variation of cases (n=133).

Seasons	n (%)
Winter	45 (33.83)
Spring	35 (26.32)
Summer	20 (15.04)
Autumn	33 (24.81)
Total	133 (100)
**Day in the week**	
Sunday	17 (12.78)
Monday	18 (13.53)
Tuesday	14 (10.53)
Wednesday	15 (11.28)
Thursday	30 (22.56)
Friday	26 (19.55)
Saturday	13 (9.77)
Total	133 (100)
**Time of the day**	
Day (08:00 to 16:00 Hrs)	97 (72.93)
Afternoon (16:00 to 22:00 Hrs)	25 (18.80)
Night (22:00 to 08:00 Hrs)	11 (8.27)
Total	133 (100)

**Figure 2:**
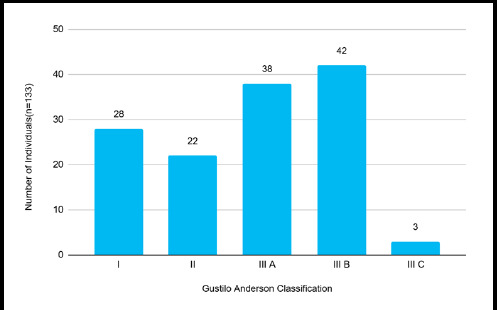
Gustilo Anderson Classification of Open Fractures (n=133).

Gustilo type III B injuries were seen in 42 (31.58%) cases followed by Gustilo type III A in 38 (28.57%) cases (Figure 2).

Among the anatomical regions, fractures involving the leg, tibia with or without fibula fractures were seen in 56 (42.11%) cases, and hand, wrist, ankle, and foot fractures were seen in 24 (18.04%) cases. Spinal anesthesia was used in 70 (52.63%) cases. Operations included external fixators 36 (27.07%), intramedullary nails 29 (21.80%), and multiple combinations 19 (14.28%), among other methods. Antibiotic usage showed single agent (Cefuroxime) used in 72 (54.14%) cases, with double and triple combinations used in 35 (26.32%) and 13 (9.77%) cases, respectively (Table 2).

**Table 2:** Details of the open fractures and their management (n=133).

**Anatomical regions**	**n (%)**
Arm - Humerus Fractures	3 (2.26)
Forearm - Radius / Ulna Fractures	20 (15.04)
Thigh - Femur Fractures	8 (6.01)
Leg - Tibia Fractures	56 (42.11)
Multiple Combination	22 (16.54)
Hand, Wrist, Ankle and Foot	24 (18.04)
**Types of anesthesia used**	
Local	3 (2.26)
Block (Brachial and other)	26 (19.55)
Spinal	70 (52.63)
GA	30 (22.56)
Combined	4 (3)
**Types of operations**	
Intramedullary nails (IMIL or rush nail)	29 (21.80)
External fixator	36 (27.07)
Ilizarov (full or hybrid)	9 (6.77)
JESS	5 (3.76)
K wire fixation	19 (14.28)
Plates and screws	8 (6.02)
Splints/slabs	8 (6.02)
Multiple combinations	19 (14.28)
**Antibiotics**	
Single (Cefuroxime)	72 (54.14)
Double (Cefuroxime + Aminoglycoside)	35 (26.32)
Triple (Cefuroxime+Metronidazole/Crystalline Penicillin + Amikacin)	13 (9.77)
Documents could not be traced	13 (9.77)

* K Wire: Kirshner; JESS: Joshi External Stabilization System

Mode of injury included Road traffic accidents causing 53 (39.85%) injuries and falls (32, 24.06%). Definitive management was immediate in 42 (31.58%) cases and within 48 hours in 48 (36.09%) cases. The discharge occurred within a week for 83 (62.41%) and in one to two weeks for 26 (19.55%) cases (Table 3). Intensive care unit (ICU) management was needed in 7 (5.26%), 22 (16.54%) of patients had hemoglobin less than 10g/dl at presentation, and 17 (12.78%) patients needed at least one pint of blood (or blood product) transfusion.

**Table 3:** Injury parameters and timelines of Open Fractures (n=133).

Mode of injury	n (%)
Road traffic accidents	53 (39.85)
Fall-related injuries	32 (24.06)
Workplace accidents	16 (12.03)
Assaults	8 (6.02)
Documents could not be traced	24 (18.04)
**Time to definite management**	
Immediately	42 (31.58)
Within 24 hours	28 (21.05)
Within 48 hours	20 (15.04)
Beyond 48 hours	19 (14.29)
Documents could not be traced	24 (18.04)
**Time to discharge from hospital**	
Within one week	83 (62.41)
One to two weeks	26 (19.55)
Two to three weeks	8 (6.01)
Documents could not be traced	16 (12.03)

## DISCUSSION

The majority of patients presenting with open fractures at our center were male (78.6%). There is probably a preponderance of open fracture in males, however, as this was a cross-sectional study, the design and the sample of the study might not be sufficient to prove male preponderance. Nevertheless, there are studies^3-6^ demonstrating male preponderance in case of open fracture, therefore it is highly likely that our finding represents the true picture. The median age of presentation in our study was 35.00 (25.00 - 42.00) years which was similar to 36.4±12.2 years reported by Odatuwa-Omagbemi^3^ Similar results in the age presentation may be because in both studies, the major mechanism of injury is RTA which affects the active population. Similar to other studies, male (78.6%) preponderance was found in our study.^3-6^

The highest number of presentations were in the second half of the week, during the daytime, and in winter seasons, similar to a study by Da Costa et al.^7^ However, in another study in Brazil, Arruda LR et al. showed the presentation was maximum during nighttime.^8^ The reason for the discrepancy could be because a significant portion of the sample reported the use of alcoholic beverages and drugs in a study by Arruda et al. where the presentation was maximum during night-time.^8^ In the Nepalese context, the 'No Drinking and Driving' policy initiated by the Metropolitan Traffic Police in Kathmandu has reduced casualties from RTAs which can lead to decreased cases, especially during night time.^9^

The most common fracture was of the tibia, which may be because the tibia is subcutaneous along its whole length in its anteromedial border.^4,10^ More than half of the cases were Gustilo type III open fractures, which were the most common (Type III B was the commonest), in line with other studies from developing countries.^3,5,7^ Our center's location at the intersection of two major highways and the lack of other tertiary care facilities nearby are likely the causes of the high number of Gustilo III open fractures we receive. In addition to having trained staff and advanced surgical capabilities, our center also offers robust prehospital services. ^3^

The use of antibiotics is warranted in all cases of open fractures as the risk of infection varies, spanning from 0% to 2% for Type I fractures, 2 to 10% for Type II fractures, and 10 to 50% for Type III fractures.^11-13^ Administering a cephalosporin for gram-positive coverage within one hour of injury provides a benefit for preventing infection in open fractures.^14^ Cefuroxime was given to more than half of the cases (54%) in our study. This antibiotic has been the choice in our center because of its broader spectrum coverage of gram-positive and negative activity as well as good bone penetration and concentration. Depending upon the degree of contamination and wound status, our institutional protocol is to add Aminoglycosides and or anaerobic coverage whenever relevant. ^15^

The methods for the fixation of open fractures have evolved, yet remain controversial.^16^ In our study, external fixators are most frequently used because the majority of cases were Gustilo III B type and external fixation provides mechanical stability to fracture while soft tissue care could be done, and prevents the risk of added infection by avoiding the need for insertion of implants directly into the injured area.^17^ The definitive management was done immediately (just after emergency room resuscitation was completed) in 31% of cases and within 24 hours in 21% of cases. The time for definitive management of open fracture was comparatively more in our case than in the study by Harley et al.^18^ The reason for the delayed definitive management in our setup can be attributed to logistic constraints and, a number of socio-economic factors such as consent and financial clearance.

About two-thirds of the cases of open fractures were discharged from the hospital within one week which is a shorter period than a similar study done in Nigeria. The study in Nigeria ascribed the longer length of hospital stay to infections, higher grade of injury, and scarcity of resources.^3^

Open fractures are viewed as having an increased risk of infection, nonunion, bone loss, and complications related to soft tissue.^19^ Complex injuries like open fractures have a high rate of morbidity and mortality. The most crucial complication of open fractures is the risk of wound infection and prompt surgical irrigation and debridement are the mainstay of prevention.^20^ Hence, standard treatment protocols must be followed to manage open fractures. Our management protocol aligns well with the standard guidelines regarding surgical management and use of antibiotics, etc.

As this is a retrospective study some of the information could not be traced. The study relies on retrospective data from hospital records which may be subjected to inaccuracies or incomplete information. Also, there may be variations in how different personnel can record data differently which can affect the study's results. No follow-ups were done (cross-sectional study design) hence cause / effect and outcome could not be analyzed.

## CONCLUSIONS

The epidemiological patterns, temporal distribution, injury pattern, and pattern of management for open fractures in our institution were similar to other studies in the literature. The frequency of the male population with open fractures was higher as compared to females. Younger populations were frequently involved and RTA was the most common cause for open fractures. The anatomical site most affected was the tibia, and Gustilo Type III B fractures were most commonly seen. Most cases had presented in the winter season. External fixator application was the most common procedure for immediate stabilization.
